# Supplementary material for: Social network determinants of alcohol and tobacco use: A qualitative study among out of school youth in South Africa
Source: PLoS One. 2020 Oct 20;15(10):e0240690. doi: 10.1371/journal.pone.0240690 (PMC7575104; doi:10.1371/journal.pone.0240690)
Supplement: S1 File — (DOCX) [file pone.0240690.s001.docx]

***Document 8***

**SEMI STRUCTURED DISCUSSION GUIDE FOR SCHOOL DROPOUTS (IN-PERSON AND WHATSAPP INTERVIEW)**

1. **Ice Breaker:**

What type of phone do you have?

What do you like to do for fun? (Probe further)

Where do you live?

How old are you?

Do you have any siblings?

How do you usually communicate with your friends?(Probe: why do you prefer that method?)

Are you male or female? (Only applicable for WhatsApp interviews if it is not clear)

How do you know your recruiter? (Only applicable if recruited into the study by a participant)

How did they tell you about the study? Did they phone, SMS, WhatsApp etc.? (Only applicable if recruited into the study by a participant)

1. **Personal experiences with smoking and alcohol use**

2.1 What do you think about smoking or using tobacco?

2.2 What do you think about drinking alcohol?

2.3 We hear many young people smoke and/or drink? What about you?

If answered yes to question 2.3:

2.3.1 What made to start?

2.3.2 Tell me about your first experience smoking a cigarette and/or drinking alcohol (Probe: age of initiation, with whom, feelings of smoking)

2.3.3 How many cigarettes do you smoke in one week?

2.3.4 How many drinks do you have in one week?

2.3.5 Why do you still smoke and/or drink?

2.3.6 Where do you get your cigarettes and/or alcohol from?

2.3.7What do you like about smoking and/or drinking? (Probe: type of drinks, type of tobacco use)

2.3.8 What do dislike about smoking and/or drinking? (Probe: Why)

2.3.9 How easy or difficult do you think it would be to stop smoking and/ or drinking? What makes it easier or harder?

If answered no to question 2.3:

2.3.7 Have you ever tried smoking/using tobacco

Have you ever tried alcohol in your life?

If answered yes to question 2.3.7:

2.3.7.1 What made to start?

2.3.7.2 Can you tell me about your first experience smoking and/or drinking? (Probe age of initiation, type of drinks, and type of tobacco use)

2.3.7.3 How many cigarettes did you smoke in one week?

2.3.7.3 How many drinks would you have in one week?

2.3.8 Can you tell me why you quite/ never tried smoking and/ or drinking?

2.3.9 What do you think is good about smoking and/or drinking?

2.3.10 What do you think is bad about smoking and/or drinking?

2.3.11 How easy or difficult was it to stop or to never smoke and/ or drink? What made it easy or difficult?

1. **Environmental experiences with smoking and alcohol and use**

*Family*

3.1 Can you tell me about your family (probe: How close are you with your family, family structure, currently residing with family, type of dwelling?)

3.2 How is your family’s smoking and/or drinking? (probe: frequency)

3.3 Can you tell me why you think your family smoke and/or drink?

3.4 If answered yes to smoking and/or drinking (question 2.3): How is your smoking and/or drinking at home?

3.5 In your opinion, what role does your family play in your decision to smoke and/ or drink?

*Friends*

3.6 Can you tell me about your close friends? (Probe age, gender, how do you know them, in – school or out of school, what do they usually do when together)

3.7 How is your friends smoking and/or drinking? (Probe: frequency, alcohol and smoking status of friends)

3.8 Can you tell me why your friends smoke and/or drink?

3.9 In your opinion, what role do your friends play in your decision to/not to smoke and/ or drink? (probe: do they pressure you, how easy or difficult is it to stay away from drinking or smoking)

3.10 What information do you know or have been told about smoking and drinking by your family or friends?

*Others*

3.6 Can you tell me about other people you are close to? (Probe age, gender, how do you know them, in – school or out of school, nature of relationship)

3.7 How is your [other person mentioned above] smoking and/or drinking? (Probe: frequency, alcohol and smoking status of other)

3.8 Can you tell me why your [other person/ people mentioned above] smoke and/or drink?

3.9 In your opinion, what role do your [other person/ people mentioned above] play in your decision to/not to smoke and/ or drink?

3.10 What information do you know or have been told about smoking and drinking by your [other person/ people mentioned above]?

1. **Reasons for leaving school**

*Personal*

1. How many people do you know who have dropped out of school, they are between13-20 years, not enrolled in a primary or high school for the thisyear and have not completed their matric?
2. Can you tell me why you left school? (Probe: last grade participant was in, school achievement, dropout age)
3. How do you spend your time now?
4. How has leaving school been good for you?
5. How has leaving school not been so good for you?
6. How has leaving school changed the way you drink and/ or smoke? (Probe: frequency, type of tobacco and alcohol use)

If yes to 4.6

4.6.1 How many drinks do you have in one week compared to before you left school?

4.6.2 How many cigarettes do you smoke in one week compared to before you left school?

4.6.3 What made did you change your drinking and/or smoking behaviour once you left school?

*Family*

1. How do your parents feel about you leaving school? (Probe: did your parents complete school, what do they do now?)
2. How does the rest of your family feel about you leaving school? (Probe: number of family members who are school dropouts)
3. What role did your family play in your decision to leave school?

*Friends*

4.10 How do your friends feel about you leaving school? (probe: Why do you think they feel this way?number of friends who are school dropouts?)

4.11 What role did your friends play in your decision to leave school?

*Others*

Are there any other people in your life that played a role in your decision to leave school

4.12 How do other people you did not mention already feel about you leaving school? (Probe: did they complete school, what do they do now?)

1. **Method Evaluation**

IM interview:

1. How do you feel about this interview being done over WhatsApp?
2. What did you like about this interview?
3. What did you dislike about this interview?
4. Did you understand the questions?
5. What made it difficult for you to do his interview over WhatsApp?
6. What made is easy for you to do this interview on WhatsApp?
7. How would you feel if we did this interview in-person?
8. Can you suggest other ways we can improve this interview?

In-person interview:

1. How do you feel about this interview being done in-person?
2. How would you feel if we did this interview on WhatsApp?
3. What did you like about this interview?
4. What did you dislike about this interview?
5. Did you understand the questions?
6. What made it difficult for you to do his interview in-person?
7. What made is easy for you to do this interview in-person?
8. Can you suggest other ways we can improve this interview?
9. **Closing**

Thank you, this is the end of the interview.

For face-face interviews

For participating in the interview, here is money for transport.

For WhatsApp interviews:

For participating in the interview, here is your voucher code to get data. To protect your identity and the answers you gave in the interview, please will you delete this chat now.

1. **PEER RECRUITMENT PROCEDURE**

Please could you assist me in recruiting your friends and family to participate in the study that also did not complete school? They have to be between the ages of 13 – 20, have not completed school and have not been enrolled in school this year. I need three people like this. If they are interested in taking part in the study, please tell them to call or message me on __________________________

Would you like to write this number down?

Here is more information on the study that you can give to your recruiters. (*hand over information sheet or send information sheet on WhatsApp)*

For every person you get to participate in the study, you will receive a R20. I would really appreciate your help.

Thank you.
